# Using cross-recurrence quantification analysis to compute similarity measures for time series of unequal length with applications to sleep stage analysis

**DOI:** 10.1038/s41598-024-73225-x

**Published:** 2024-10-04

**Authors:** Henning Johannes Drews, Flavia Felletti, Håvard Kallestad, Annika Drews, Jan Scott, Trond Sand, Morten Engstrøm, Hanne Siri Amdahl Heglum, Daniel Vethe, Øyvind Salvesen, Knut Langsrud, Gunnar Morken, Sebastian Wallot

**Affiliations:** 1 Department of Mental Health, Norwegian University of Science and Technology, Trondheim, Norway; 2 Institute for Sustainability Education and Psychology, Leuphana University of Lüneburg, 21335 Lüneburg, Germany; 3 Department of Mental Health Care, St. Olavs University Hospital, Trondheim, Norway; 4 Copenhagen, Denmark; 5 Institute of Neuroscience, Newcastle University, Newcastle upon Tyne, UK; 6 Department of Neuromedicine and Movement Science, Faculty of Medicine and Health Sciences, Norwegian University of Science and Technology (NTNU), Trondheim, Norway; 7 Department of Neurology and Clinical Neurophysiology, St Olavs University Hospital, Trondheim, Norway; 8 Department of Public Health and Nursing, Norwegian University of Science and Technology, Trondheim, Norway; 9 Independent researcher, Stuttgart, Germany

**Keywords:** Cross-recurrence analysis, Mortality, Sleep cycle, REM/NREM cycle, Sleep regularity, Human behaviour, Risk factors, Statistics

## Abstract

Comparing time series of unequal length requires data processing procedures that may introduce biases. This article describes, validates, and applies Cross-Recurrence Quantification Analysis (CRQA) to detect and quantify correlation and coupling among time series of unequal length without prior data processing. We illustrate and validate this application using continuous and discrete data from a model system (study 1). Then we use the method to re-analyze the Sleep Heart Health Study (SHHS), a rare large dataset comprising detailed physiological sleep measurements acquired by in-home polysomnography. We investigate whether recurrence patterns of ultradian NREM/REM sleep cycles (USC) predict mortality (study 2). CRQA exhibits better performance compared with traditional approaches that require trimming, stretching or compression to bring two time series to the same length. Application to the SHHS indicates that recurrence patterns linked to stability of USCs are associated with all-cause mortality even after controlling for other sleep parameters, health, and sociodemographics. We suggest that CRQA is a useful tool for analyzing categorical time series, where the underlying structure of the data is unlikely to result in matching data points—such as ultradian sleep cycles.

**Supplementary Information:**

The online version contains supplementary material available at 10.1038/s41598-024-73225-x.

## Introduction

A major challenge to quantifying similarities or relationships between two time series or sequences is that time-bound processes often do not have the same duration. This is particularly conspicuous for real-life phenomena, ranging from transcripts of conversations^1^, writing texts and typing^2^, fixations and scan-paths of eye movements^3^, through to physiological processes, such as RR-intervals of different people^4^ or the duration of sleep episodes^5,6^. To correlate such data series, an equal amount of time-matched data points is needed. The standard approach to ensuring that data series conform to this requirement includes either trimming, stretching or compressing of the data series^3,7^. Stretching or compressing can be achieved by specific procedures such as re-sampling or interpolation. However, these strategies presuppose that the implicit or explicit modeling assumptions of these procedures adequately reflect the true dynamics or sequential properties of the data. In other words, they presuppose a relatively thorough a priori understanding of the temporal dynamics of data, which is often not available. If the temporal structure of the data does not meet these assumptions, trimming, re-sampling, and interpolation procedures can lead to the introduction of spurious correlations or the exclusion of existing correlations^7^, making similar time series appear very different (or vice versa). This problem is even more prominent for the analysis of nominal sequences, as re-sampling or interpolation procedures are unfeasible for this kind of data. Thus, there is a need for a robust method to compare timeseries of unequal length, ideally independent of data type (i.e., including nominal data).

The relevance of sleep for human health is attracting growing attention^8^. However, a comprehensive sleep assessment requires cardiorespiratory polysomnography (PSG) a costly and labour-intensive technique to simultaneously monitor multiple physiological parameters (such as EEG, ECG, EOG, EMG)^9^. Thus, only a limited number of large datasets including polysomnographic measurements exists. Moreover, even if PSG is conducted, the analysis of sleep typically focuses on sums, averages, or ratios across the entire night (e.g., total duration of sleep stages). This fails to take into account findings from basic sleep research indicating that the sequential order of sleep stages is functionally important^10^. For instance, optimal memory consolidation depends on sequential deep sleep and REM sleep^10^. A fundamental mode of sequential ordering of sleep stages are ultradian NREM/REM sleep cycles (USCs). USC are sequences of nominal data (i.e., sleep stages) that oscillate on an ultradian frequency of approximately 4 (3.9 ± 1.23^11^) per night with a period of approximately 120 min (117 ± 39 min^11^). A very limited body of small-sample basic research studies indicates that changes in individual sleep cycles might be differentially associated with sleep and health outcomes^12–15^. This calls for a deeper investigation of USC similarities and their connection with health.

Yet, as USCs usually differ in length, their detailed longitudinal comparison requires overcoming the above-described methodological problem of comparing time series of unequal length.

In this article, we aim to investigate USC similarities, i.e., specific recurrence patterns of sleep stage sequences between USCs and their association with all-cause mortality by re-analyzing a rare large dataset of PSG data derived from the publicly available Sleep Heart Health Study (SHHS;^16,17^). In order to do so, we introduce and validate a method that offers a solution to the problem of unequal time series, which is Cross-Recurrence Quantification Analysis (CRQA).

Here, we extend classical CRQA^18^, a nonlinear bivariate correlation technique to discover correlation and coupling between two time series, by relaxing the constraint that time series subjected to this method need to have the same number of data points. Moreover, this application of CRQA is readily applicable to nominal sequences of different length and enables computation of recurrence-based similarity measures^19^.

**Fig. 1:**
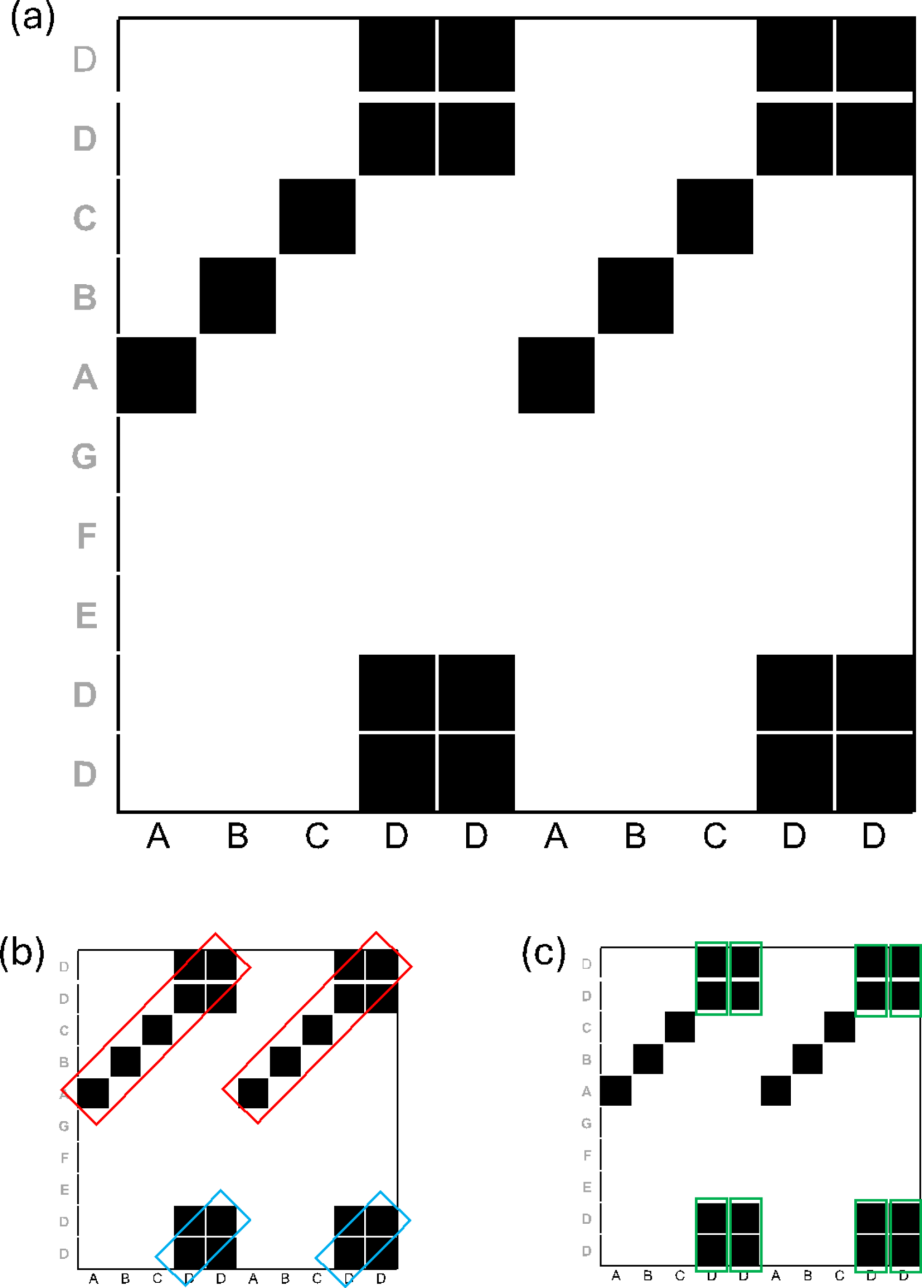
Example CRP and line structures on the plot. (**a**) Two example nominal sequences: One is plotted on the x-axis and one is plotted on the y-axis. If values from one series appear in the other series, these values are counted as cross-recurrences between the series, marked by black squares. From the recurrence plot, quantifiable measures can be derived that either are global measures of similarity between the times series (e.g., recurrence rate) or capture similarity on a trajectory level (e.g., determinism or diagonal line lengths). (**b**) Highlight of diagonal lines of length 5 in red and diagonal lines of length 2 in blue. (**c**) Highlight of vertical lines of length 2 in green. Example taken from Leonardi and Wallot^20^, with permission.

Simply put, cross recurrence plots (CRPs) are a means of depicting how patterns in one time series (or sequence) are repeated in another time series. Such repetitions can be absent, in which case there is no correlation between the two time series (they are very dissimilar). While individual cross-recurrences between two time series indicate that particular data points (or coordinates) which occur in one time series also occur in the other, extended patterns of recurrences show that there are whole sub-sequences in terms of which the two time series exhibit similarity. We will illustrate this graphically (Fig. 1), show how one can derive quantitative measures of similarity from such cross-recurrence plots—and also how this can be done for time series of unequal length or non-matching data points.

CRQA is amendable to different types of data, this includes series of nominal data, in which we are primarily interested in the current paper. The basis of CRQA is the Cross-Recurrence-Plot (CRP), which charts recurrent values between two time series—or sequences. In the method section of this paper, we provide the basic equations that are used to construct Cross-recurrence Plots, but they are easily illustrated using a simple example: Imagine two nominal sequences, “ABCDDABCDD” and “DDEFGABCDD”. If we are interested in how these two sequences are similar, also in terms of their order (or temporal evolution), we can view them as a CRP (Fig. 1).

On this CRP, dark squares indicate cross-recurrences, and white surfaces the absence of recurrence. For example, we see that the sequence plotted on the y-axis contains the sub-sequence “DD” at the beginning (and end). Accordingly, there are four squared (recurrence) where the first sub-sequence “DD” from the y-axis also appears on the x-axis (after the “ABC”), the presence of this pattern in both sequences. Besides this visual display, CRQA also allows to quantify the similarity of two sequences. Here, several measures have been developed^21^. A first, frequently used basic measure that gives a global impression of the similarity of two time series is the %REC, the cross-recurrence rate (or total recurrence). The cross-recurrence rate is simply calculated as the sum of all cross-recurrence points (black squares in Fig. 1a) divided by the size of the CRP (in this case: 22/100 = 0.22 or 22%). More fine-grained recurrence measures focus on diagonal line structures in the recurrence plot. Diagonal line structures represent trajectories that recur in both time series (Fig. 1b). Some of the most common ones are:

%DET (“percent determinism”), which is the number of cross-recurrence points on the CRP that have at least one diagonal neighbor (from lower-left to upper-right) divided by the sum of all recurrence points. On the plot above (Fig. 1), we see the two long diagonals in the upper half of the plot, which are diagonals of length 5, as well as the two squares on the bottom, which contain diagonals of length 2. For this example plot, there are thus four diagonal lines of recurrence points of lengths 5, 5, 2, and 2 (Fig. 1b). Hence, there are 14 recurrence points with at least one diagonal neighbor. Accordingly, %DET is 14/22 = 0.64 or 64%. %DET is a measure of how much the similarity of the two sequences is structured in terms of repeating sub-sequences.

%LAM (“percent laminarity”) is similarly computed but is based on vertical line structures on the CRP. Specifically, the number of cross-recurrence points that have at least one vertical neighbor divided by the sum of all recurrence points. On the plot above (Fig. 1), we see four 2-by-2 squares of recurrence points (Fig. 1c). Hence, there are 16 recurrence points with at least one vertical neighbor. It follows that %LAM is 16/22 = 0.73 or 73%. %LAM is a measure of how much the similarity of the two sequences is structured in terms of “states”, where identical or very similar values are repeated back-to-back (here, the letter “D”).

Finally, another common measure is ENTR (“entropy”), which is the Shannon Entropy of the distribution of diagonal lines. As we have seen in the calculation of %DET for the CRP depicted in Fig. 1b, this distribution contains four diagonal lines: Two lines of length 5 and two lines of length 2. The Shannon entropy is now calculated as the negative sum of the logarithm of the relative frequency with which different lines lengths are occurrence, that is: -log(2/4) + -log(2/4) = 0.30 + 0.30 = 0.60. Here, the frequencies and their distribution come from the distribution of diagonal lines on the CRP. Hence, this is a measure of the complexity of the shared cross-recurrence patterns between the two sequences.

In the case of sleep measurements, a high degree of reoccurring diagonal line structures indicates that sleep time series are similar with respect to their timely sleep-stage trajectories. Finally, one can also quantify vertical and horizontal line structures. These phenomena indicate that one of the time series lingers in a specific state (e.g., a certain sleep stage) for a comparably longer time than the other. In the following, we want to show how to apply CRQA to calculate similarity measures of nominal sequences, particularly for cases where the number time data points in each sequence does not match.

In this work, we present two studies:

The first study is a simulation study in artificial datasets validating CRQA against conventional linear correlations including trimming and stretching/compression techniques. The second study uses CRQA to re-analyses the SHHS dataset in order to investigate recurrence patterns of USCs and their predictive power regarding mortality.

We hypothesize that (1) CRQA is superior to standard approaches to correlate longitudinal data independent of data type (i.e., continuous data or categorical data); and (2) recurrence patterns of USCs predict mortality even after controlling for conventional sleep data and other covariates.

## Results

This publication aims to improve the comparison of time series of unequal length by using CRQA, a method that can overcome the requirement of matching data points that is present in many methods to compare time series. Moreover, we use CRQA to analyze USC similarities and explore their association with all-cause mortality.

In detail, we present two studies. A simulation study comparing CRQA to frequently used other methods to compare time series (study 1) and a re-analysis of a large polysomnographic dataset—the Sleep Heart Health Study^16,17^; study 2).

### Study 1: a simulation study comparing CRQA vs. Pearson correlation and Cramer’s V correlation

The first study aimed to compare CRQA (for a detailed description of the method please see the Method section) and conventional methods with respect to correlating time series of unequal length.

**Fig. 2:**
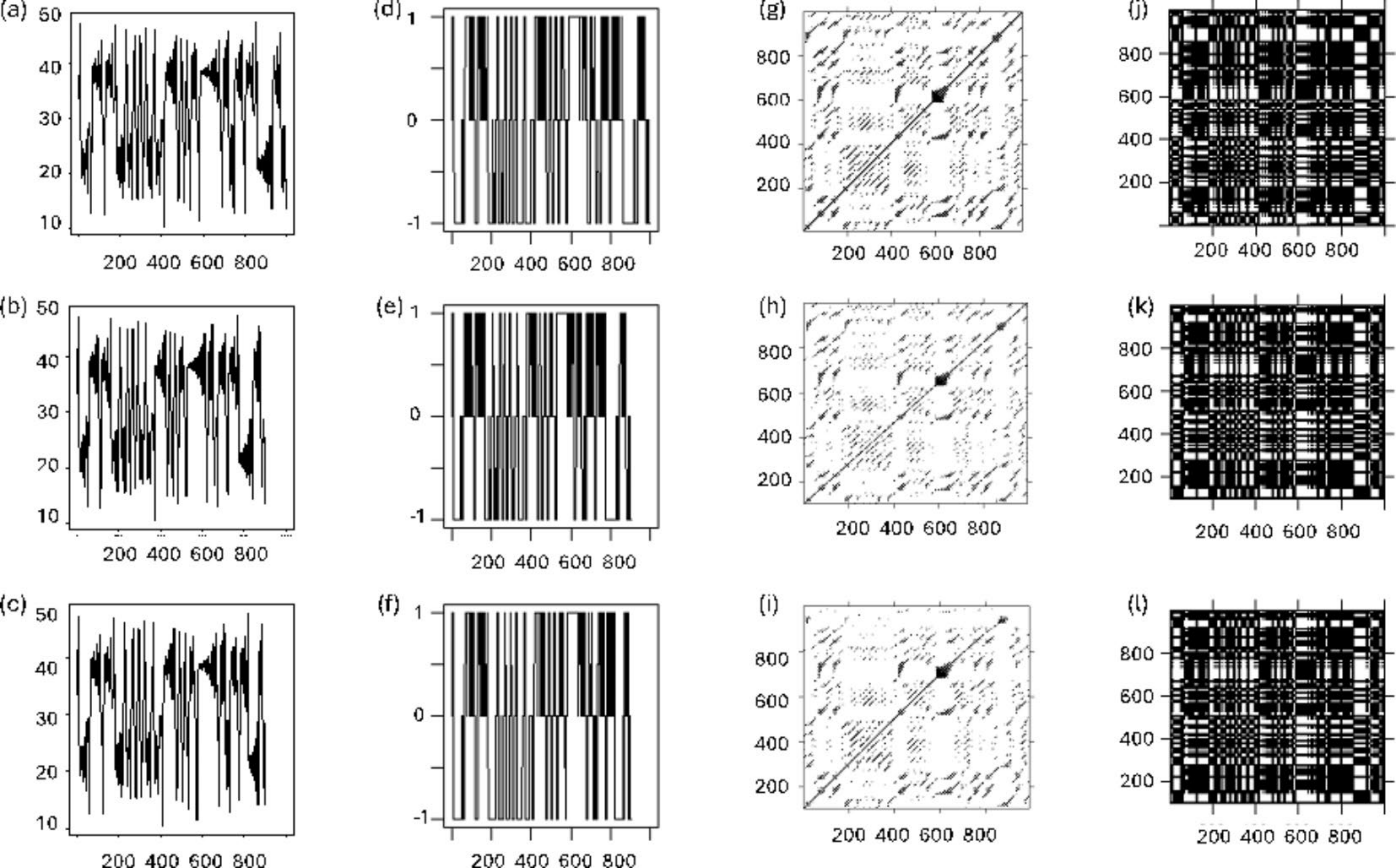
Example time series. (**a**) s_ori (i.e., the original data), continuous (1000 data points), (**b**) s_lin, continuous (linearly compressed data—900 data points), (**c**) s_exp, continuous (exponentially compressed data—900 data points), (**d**) s_ori, categorical (1000 data points), (**e**) s_lin, categorical (linearly compressed—900 data points), (**f**) s_exp, categorical (exponentially compressed—900 data points). Selected example cross-recurrence plots: (**g**) Cross-recurrence plot of data s_ori with itself, but with different instantiations of the noise component (continuous case); (**h**) Cross-recurrence plot of data s_ori with data s_lin (continuous case); (**i**) Cross-recurrence plot of data s_ori with data s_exp (continuous case); (**j**) Cross-recurrence plot of data s_ori with itself, but with different instantiations of the noise component (categorical case); (**k**) Cross-recurrence plot of data s_ori with data s_lin (categorical case); (**l**) Cross-recurrence plot of data s_ori with data s_exp (categorical case).

Therefore, artificial datasets of continuous and categorical data of unequal length were created. Point of departure were continuous and discretized data with added noise derived from the x-dimension of the Lorenz attractor^22^, Fig. 2a&d). These original datasets were compressed linearly and exponentially (Fig. 2b-c&e-f). From these artificial datasets, cross-recurrence plots were calculated (Fig. 2g–l). When comparing the different cross-recurrence plots visually, it already becomes apparent that they are qualitatively very similar, despite the fact that the cross-recurrence plots in Fig. 2h–I and k–l are computed on time series of different length, and with distorted dynamics with regard to one another. In the following, we will examine in how far this visual similarity is also reflected in the quantification of the plots.

Next, we tested the performance of CRQA and conventional methods when correlating our artificial time series of unequal length. The conventional methods tested were Pearson correlation for continuous data and Cramer’s V^23^ for categorical data. Both methods require two series of same length. Two basic data pre-processing techniques were tested, which are used to deal with the problem of correlating two time series of different length: Trimming and resampling. For trimming, we trimmed the beginning of the longer of the two series or alternatively performed trimming from the end. Trimming is a simple procedure that makes two time series match in terms of the number of data points, but that usually has very adverse consequences for the alignment of the data points in time: The trimmed data points from the longer time series are usually those that should be matched with corresponding data points from the shorter time series, but are now lost due to their removal. Accordingly, trimming runs a high risk of ending up with time series of non-matching data points, artificially removing any real correlation between the time series.

A more fitting method is compression through resampling of the longer time series (or, conversely, interpolation of the shorter time series). However, one needs to choose a function or calculation procedure that makes assumptions about the process that produced differences in length between the two time series. Thus, the success of resampling relies on whether these assumptions are met, and whether the researcher possesses sufficient knowledge about the process to pick a proper resampling procedure. In the following, we will use linear resampling, as this is the default that is generally used in the absence of more specific knowledge of the phenomenon at hand.

Looking at the results, all of these procedures produce correlation coefficients substantially below the expected value for time series of equal length (original with itself, but different noise, Table 1), and all show substantial bias, as described by the Δ-values, relating the difference of each of the trimmed cases to the ground truth (Table 1). There is one exception, and that is the case “s_ori, s_lin”, where the shorter time series, s_lin, is simply a linearly compressed version of s_ori (plus noise). If the longer time series is linearly down sampled, there is basically a perfect match (compared with the expected values). However, if the compression is not linear (as for “s_ori, s_exp”), inaccuracies appear again. This is noteworthy because many analyses of behavioral and physiological data often assume (as a default assumption) that there is a linear relationship between two variables.

**Table 1:** Pearson’s *r* and Cramer’s *V* correlations (with 95% confidence intervals) for time series of equal and unequal length that have been trimmed or compressed to meet the criterion of matched data points.

	Continuous		Categorical	
	*r*	Δ*r*	*V*	Δ*r*
s_ori, s_ori	0.801 [0.784, 0.817]	–	0.553 [0.525, 0.583]	–
s_ori, s_lin (front)	0.032 [0.004, 0.062]	0.385	0.081 [0.048, 0.114]	0.472
s_ori, s_lin (back)	0.026 [−0.003, 0.055]	0.388	0.086 [0.054, 0.116]	0.467
s_ori, s_lin (linear)	0.800 [0.783, 0.818]	< 0.001	0.552 [0.525, 0.584]	0.001
s_ori, s_exp (front)	0.483 [0.449, 0.517]	0.156	0.334 [0.301, 0.364]	0.219
s_ori, s_exp (back)	0.053 [0.021, 0.098]	0.374	0.060 [0.023, 0.097]	0.91
s_ori, s_exp (linear)	0.043 [0.041, 0.046]	0.379	0.036 [0.020, 0.055]	0.517

Note. *R* = Pearson correlation coefficient, ranging from − 1 to 1; *V* = Cramer’s *V* correlation coefficient, ranging from 0 to 1. Δ = difference between the average correlation of time series of equal length (s_original with itself, but different of the noise term) compared with the specific trimmed case, divided by the range of the scale of the respective correlation coefficient. Front = cutting off the first data points of the longer time series to achieve equal length of both series. Back = cutting of the last data points from the longer time series to archive equal length of both series. Linear = Linearly down sampling of the longer time series to yield equal length of both series.

CRQA does not need the two-time series to be of equal length. We can enter the different series directly into the analysis, without trimming or compressing.

The selected cross-recurrence measures show substantially less bias than found when applying cutting and resampling procedures (as described above). Notably, in the analysis of the categorical data, the 95% confidence intervals between the original time series and the unequal time series overlap. The same holds true for continuous data except for % total recurrence (Table 2). Accordingly, cross-recurrence analysis seems much more appropriate in cases where two time series do not have the same length or matching data points and can handle such data even without pre-processing that resamples, interpolates or trims time series to meet these criteria.

**Table 2:** RQA outcome measures (with 95% confidence intervals) for time series of equal and unequal length.

	%REC	%DET	%LAM	Δ %REC	Δ %DET	Δ %LAM
Continuous						
s_ori, s_ori	0.19 [0.18, 0.21]	1.25 [0.53, 1.9]	2.39 [0.95, 4.29]	–	–	–
s_ori, s_lin	0.14 [0.13, 0.15]	0.91 [0.17, 1.780]	1.74 [0.48, 3.52]	< 0.001	0.003	0.006
s_ori, s_exp	0.14 [0.12, 0.15]	0.91 [0.17, 1.75]	1.70 [0.46, 3.44]	< 0.001	0.003	0.007
Categorical						
s_ori, s_ori	41.64 [41.17, 42.16]	96,72 [96,14, 97,25]	98,79 [98,22, 99,29]	–	–	–
s_ori, s_lin	41.56 [40.63, 41.59]	96,56 [95,96, 97,08]	98,76 [98,20, 99,26]	< 0.001	0.002	< 0.001
s_ori, s_exp	41.09 [40.63, 41.93]	96,47 [95,88, 97,03]	98,69 [98,12, 99,20]	0.006	0.003	0.001

Note. *%REC* = percent recurrence, ranging from 0 to 100; *%DET* = percent determinism, ranging from 0 to 100. *%LAM* = percent laminarity, ranging from 0 to 100. Δ = difference between the average cross-recurrence measure of time series of equal length (s_ori, s_ori) compared to the specific cases of time series with unequal length, divided by the range of the scale of the respective correlation coefficient. Embedding parameters for the continuous data were: delay = 3, embedding dimension = 3, threshold = 0.1, normalization = “euc”. Embedding parameters for the categorical data were: delay = 1, embedding dimension = 1, threshold = 0.0001, normalization = “euc”.

### Study 2: using CRQA to study sleep-related mortality in the SHHS data set

We used CRQA to reanalyze a comparably large, longitudinal cohort of elderly (> 60 years) individuals (*N* = 2248, age 72.1 ± 7.1 years, male = 48%, for sample characteristics see Table 3) from whom neurophysiological sleep data was obtained by at-home-polysomnography (PSG). This sample was derived from the Sleep Heart Health Study, a multi-state prospective study in the US^16,17^.

**Table 3:** Sample characteristics.

	Overall
	(*N* = 2248)
Gender	
Male	1068 (47.5%)
Female	1180 (52.5%)
Age (baseline, years)	
Mean (SD)	72.1 (7.11)
Race	
White	1990 (88.5%)
Black	222 (9.9%)
Other	36 (1.6%)
BMI (baseline)	
Mean (SD)	27.7 (4.72)
SF-36 physical health	
Mean (SD)	45.5 (10.0)
SF-36 mental health	
Mean (SD)	53.8 (8.06)
Total sleep time (min)	
Mean (SD)	351 (60.6)
Sleep efficiency (%)	
Mean (SD)	80.6 (10.2)
S1 sleep (%of sleep time)	
Mean (SD)	5.62 (4.11)
S2 sleep (%of sleep time)	
Mean (SD)	57.2 (12.1)
SWS sleep (%of sleep time)	
Mean (SD)	18.0 (12.5)
REM sleep (%of sleep time)	
Mean (SD)	19.2 (6.03)
Apnea-Hypopnea-Index (AHI)	
Mean (SD)	16.1 (15.0)
# of USCs	
Mean (SD)	3.55 (1.05)
Duration of USCs (min)	
Mean (SD)	116 (32.9)

Note. SF-36 = short form survey 36; S1, S2 sleep = sleep stages as defined by Rechtschaffen&Kales^24^, SWS = Slow-wave sleep: combination of Rechtschaffen and Kales sleep stages S3 & S4, USC = Ultradian NREM/REM sleep cycle.

In detail, we investigated cross-recurrence patterns of ultradian NREM/REM sleep cycles (USC) and their association with all-cause mortality.

Overall, 846 individuals died over a median (Q1,Q3) follow-up period of 11.4 (8.6,12.4) years. Polysomnography demonstrated that individuals experienced a mean ± SD of 3.6 ± 1.1 USC per night with a mean duration of 116 ± 33 min (Table 3). The mean ‘intra-individual’ difference between the longest and shortest completed USC was 77.4 ± 47.0 min. The intra-individual mean standard deviation of the duration of USCs within one night was 39.8 ± 28.7 min.

**Fig. 3:**
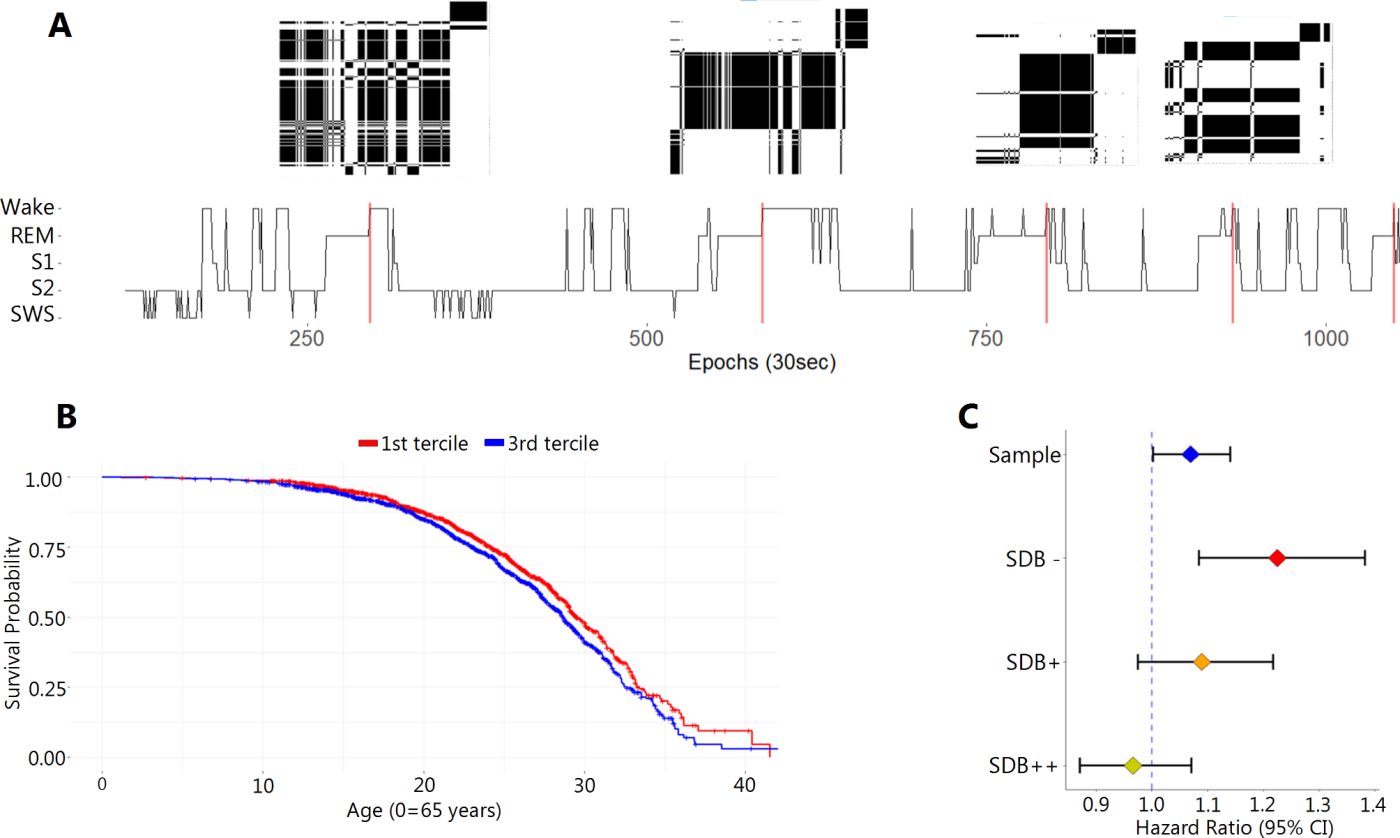
Application of CRQA to the analysis of Ultradian Sleep Cycles. (**A**) The sleep profile of one night can be subdivided into ultradian NREM/REM sleep cycles (USC, USC-endpoints = vertical red lines) of unequal length, pairwise cross-recurrence plots are calculated (exemplary recurrence plots for adjacent USC pairs (USC1&2, 2&3, 3&4, and USC 4&5) are presented on top of the upper end of the vertical red lines). Thereof, a set of CRQA parameters is derived and reduced via Principal Component Analysis. (**B**) Higher levels of the resulting CRQA-stability parameter are associated with increased mortality (red Kaplan-Meier-curve, HR: 1.09 [95% CI: 1.02, 1.16], *p* = 0.011). (**C**) The association of the CRQA-stability parameter with mortality is strongest in individuals without sleep disordered breathing (SDB –, HR: 1.22 [95% CI: 1.09, 1.38], *p* = 0.009). REM = Rapid eye movement sleep stage (dream sleep), S1 = S1 sleep stage (light sleep), S2 = S2 sleep stage (light sleep), SWS = Slow-wave sleep (combined sleep stages S3 & S4, deep sleep). SDB= Sleep Disordered Breathing.

This variability in USC length illustrates the need for addressing the unequal time series problem. Therefore, cross-recurrence plots were calculated (Fig. 3A), which can handle cases of two time series of different length.

The standard set of CRQA parameters (total recurrence, determinism, mean and maximal diagonal length, as well as entropy—which is computed as the Shannon entropy of the diagonal line lengths on the cross-recurrence plots) was calculated pairwise across all USCs of each person (see also^21^). From these calculations the mean value of all pairwise comparisons as well as the results of the comparison of USC 1 vs. USC 2 for each CRQA parameter were used for further analysis. This resulted in 10 individual parameters. The focus on the cross-recurrence patterns between USC1 and USC2 was included since converging sleep-stage ratios in USC 1 and 2 has been associated with disturbed sleep and thus might be of special importance for human health^12,13^. A principal component analysis (PCA) then reduced the number of parameters to five dimensions that accounted for 94.7% of the variance (supplementary material). A PCA parameter correlating most with the maximal diagonal length between the first and second USC and mean determinism across all USCs (herewith “CRQA-stability parameter”) proved to be positively associated with mortality (Hazard Ratio: 1.09 [95% CI: 1.02, 1.16], *p* = 0.011; Fig. 3B). This association remained statistically significant when adjusted for sociodemographic information, self-reported health, and other polysomnographic parameters that have been reported to be associated with mortality (HR: 1.07 [95% CI: 1.003, 1.14], *p* = 0.039). Sensitivity analysis for sleep disordered breathing showed that the association between the CRQA-stability parameter and mortality was strongest in individuals without sleep apnea (HR: 1.22 [95% CI: 1.09, 1.38], *p* = 0.001; Fig. 3C).

Interestingly, the only other significant sleep parameter was S1 sleep (HR: 1.02 [95% CI: 1.005, 1.04], *p* = 0.009). When excluding the CRQA-stability parameter, S1 (HR: 1.02 [95% CI: 1.004, 1.04], *p* = 0.011) and REM sleep (HR: 0.98 [95% CI: 0.98, 0.999], *p* = 0.029) were significantly associated with mortality.

Considering the current debate about the appropriate time scale of Cox-proportional hazard models in observational longitudinal datasets^25,26^, it is important to note that the association of the CRQA-stability parameter is similar when using time-on-study instead of age as time scale (HR: 1.09 [95% CI: 1.02, 1.16], *p* = 0.011; when controlling for the full set of covariates). All models complied with the assumptions for Cox-proportional hazard models.

## Discussion

This article introduces an application of CRQA to the analysis of time series of unequal length and describes its performance and utility for correlating such two time series, i.e., time series that do not have matching data points, namely by applying it to the analysis of sleep-associated mortality in the SHHS dataset.

Using the continuous and discretized data from the Lorenz system^22^, we show that the method works well even for two time series of sequences that are compressed or stretched in relation to one another. We highlight that CRQA outperforms standard approaches to continuous time series data as the latter are typically underpinned by assumptions about the structure of the data that can lead to systematic bias in the estimated parameters. This is less likely with CRQA. Moreover, CRQA can also be applied to sequences of nominal data that cannot be readily stretched, compressed, or re-sampled.

We illustrate the utility of CRQA by re-analyzing a rare large dataset of PSG data derived from the publicly available Sleep Heart Health Study (SHHS;^16,17^) by investigating recurrence patterns of NREM/REM sleep cycles (USCs) and their association with mortality. USCs are nominal sequences of different states of sleep neurophysiology, that is, sleep stages. As USCs display inter- and intra-individual variability analysis of their cross-recurrence patterns requires dealing with the problem of unequal time series.

Confirming our second hypothesis, this first-of-a-kind analysis of USC cross-recurrence patterns reports a significant association of a CRQA-stability parameter with future mortality. The finding is robust in face of other polysomnographic parameters that have been previously associated with mortality^28^ and is stronger in individuals without sleep apnea. This demonstrates that using CRQA for the analysis of sleep patterns provides a level of analysis (i.e., sequential recurrence patterns of USC) that is not amenable to conventional sleep analysis techniques and is potentially clinically meaningful.

Mechanistically, the positive association of stability with mortality seems counterintuitive. Usually, sleep instability is associated with ill-health^29,30^. However, there is a physiological sequential dynamic of sleep stages over the night. Deep sleep, for instance, is mainly present in the first USC and rapidly decreases in the following USCs^12^. Therefore, long recurrent sleep-stage trajectories between USC 1&2 could indicate a disruption of the physiological dynamics of sleep and CRQA of USC could prove useful as a measure of physiological sleep-stage dynamics. While this mechanism is speculative, the reproduction of previous works regarding number and duration of USCs in the elderly (3.6 ± 1.1 and 116 ± 33 min here vs. 3.9 ± 1.2 and 117 ± 39 min in Le Bon and colleagues^11^) as well as regarding polysomnographic predictors of mortality when not considering our CRQA parameter (i.e., S1 and REM sleep^28^), increases the overall validity and representativeness of our results for an elderly population.

Moreover, CRQA of USCs may be relevant beyond refining PSG-based sleep assessment. USC-like patterns are extractable from sleep monitoring devices that merely capture movements^31,32^. This makes the here presented method applicable for a large array of (consumer-grade) devices (e.g., actiwatches, sleep radars, fitness trackers). CRQA-derived measures might thus be among the awaited new metrics to make sense of the enormous amounts of real-world sleep data that are provided by all sorts of sleep trackers^33^.

Nevertheless, this analysis of the association of CRQA-stability and mortality has limitations. It was designed as an opportunistic analysis to demonstrate the feasibility of applying CRQA to USC and to *explore* a potential relevance for predicting mortality. Thus, several questions remain unaddressed. It remains, for instance, unclear which mechanism underlies the association of CRQA-stability and mortality and why it is stronger in individuals without sleep apnea. Clearly, a larger, structured assessment of CRQA of USC in a bigger, more diverse sample is needed.

## Methods

### Study 1: Simulation study

Study 1 is a simulation study about artificial datasets validating CRQA against conventional linear correlations including trimming and stretching/compression techniques.

#### Description of cross-recurrence quantification analysis (CRQA)

Our current application is a further extension of Cross-Recurrence Quantification Analysis (CRQA). CRQA starts with a distance matrix *D* that charts the distances between all possible pairing of data points between a time series *x* and *y* (Eq. 1):

1 $${D}_{i,j}=\|{x}_{i}-{y}_{j}\|,\quad i=1,\dots\:,N,\:\:\:j=1,\dots\:,M,\:\:\:N=M$$

Depending on the dynamics of the time series, these distances are not computed on the raw time series data, but on the phase-space profiles of the embedded time series, using time-delayed embedding procedures^34^. For introductions to phase-space reconstruction in CRQA see Abarbanel^35^.

Next, the distance matrix *D* is discretized by a threshold parameter *t* to define a Cross-Recurrence Plot (CRP) (Eq. 2):

2 $${CRP}_{i,j}={\Theta\:}\left(t-{D}_{i,j}\right),\:\:\:i=1,\dots\:,N,\:\:\:j=1,\dots\:,M,\:\:\:where\:N=M$$

Two values of the time series *x* and *y*, *x*_*i*_ and *y*_*i*_, are considered cross-recurrent (i.e., similar) if their distance |*x*_*i*_–*y*_*i*_| is smaller than a pre-defined threshold *t*, and are considered non-recurrent (i.e., dissimilar) if |*x*_*i*_–*y*_*i*_| >= *t*. For continuous data, the *t* parameter has to be set to an appropriate value (see Wallot & Leonardi^20^ for how to set threshold parameters), because continuously measured data includes measurement errors as well as endogenous fluctuations^36^. For nominal sequences, *t* is set to 0 (or a very small value), so that only identical values between *x* and *y* are counted as cross-recurrent.

After the composition of a CRP, this plot can now be quantified using different measures that capture different correlation and similarity metrics between *x* and *y*. Here, we describe three key measures: percentage recurrence (*%REC*), percentage determinism (*%DET*) and percentage laminarity (*%LAM*). (For other recurrence and RQA-measures and their computation, see Marwan et al.^21^).

Percentage recurrence (*%REC*) measures the number of recurrent points (i.e., distance values below the threshold parameter *t*) divided by the size of the recurrence plot (i.e., the number of all potentially possible recurrence points). Percent determinism (*%DET*) represents the number of recurrence points that hold a diagonal neighbor on the recurrence plot divided by the total number of recurrences, indicating how much two time series share trajectories. Percent laminarity (*%LAM*) is the number of recurrence points that hold a vertical/horizontal neighbor on the recurrence plot divided by the total number of recurrences, indicating to what extent time series share similar states. Vertical/horizontal measures are important for quantifying categorical data because these data may demonstrate clusters of recurrences rather than smooth trajectories^37^. For a description of additional recurrence measures and their computation, see Marwan et al.^21^.

CRQA can be extended to analyze time series of unequal length by relaxing the requirement that y and x need to be of the same length (i.e., *N* = *M*). Accordingly, a non-symmetric CRP can be constructed by (Eq. 3):

3 $${CRP}_{i,j}={\Theta\:}\left(t-{D}_{i,j}\right),\:\:\:i=1,\dots\:,N,\:\:\:j=1,\dots\:,M$$

While classical CRPs always form a square matrix, CRPs form rectangular matrices, where the lengths of the sides are proportional to the lengths of the time series *x* and *y*. However, such a plot can be quantified using the same cross-recurrence measures used for symmetric CRPs, and hence yield a measure of the similarity between two data series of different length without having to trim or stretch/compress these series.

An R-function for implementing the CRQA routine can be found in the online supplementary materials (see online repository for this article: https://osf.io/eygzj/).

#### Synthetic data

First, we present a comparison of CRQA to Pearson correlation as measures of similarity between two time series. We take the x-dimension of the Lorenz attractor^22^ (Eq. 4) with added noise:

4 $$\begin{aligned}\dot{x}&=\sigma\:\left(y-x\right) \\ \dot{y}&=\left(\rho\:-z\right)-y \\ \dot{z}&=xy-\beta\:z \end{aligned}$$

This simulates a signal with characteristic (deterministic) dynamics plus a source of independent (measurement) noise with a signal-to-noise ratio of 16/1. For practical purposes, we only use the time series of the x-dimension of the Lorenz system, which we will denote as synthetic data series s_original. Next, we create a linearly compressed version of s_original, which is obtained by removing every tenth data point from s_original, resulting in a linearly compressed data series s_lin. Finally, we create an exponentially compressed version of s_original, which is obtained by removing data points at exponentially increasing intervals from s_ori (i.e., the original data), resulting in an exponentially compressed series s_exp. Accordingly, all three series share characteristic dynamics, which are somewhat occluded by noise, and distorted relative to each other by linear or exponential compression.

Finally, we create nominal sequences of these data by discretizing each time series *t* into three values, −1, 0 and 1, grouping the continuous data by their terciles (*tc*) and resulting in a discrete series of values *s* (Eq. 5):

5 $${s}_{i}=\left\{\begin{array}{ll}-1&\:\:\:if\:t\le\:{tc}_{1}\\\:0&\:\:\:if\:{tc}_{1}<t{\le\:tc}_{2}\\\:1&\:\:\:if\:\:\:t\ge\:{tc}_{2}\end{array}\right.$$

#### Resampling and trimming procedures

Comparing time series of unequal length traditionally requires processing of the time series to align the data points. We undertake a trimming approach (the longer of the two series is trimmed to match the length of the shorter series by removing the respective number of data points from the beginning or end of that series). Next, we undertake linear compression (resampling the longer time series so that it yields a linearly compressed version of the original series, matching the number of data points in the shorter series).

#### Data analysis approach

Study 1 aims to compare the performance of CRQA and conventional methods by comparing original artificial time series to each of its compressed versions that have been processed by front or end trimming and linear compression. The conventional methods tested were Pearson correlation for continuous data and Cramer’s V^23^ for categorical data.

These correlations were calculated for each combination of the original dataset with either the linearly compressed or exponentially compressed derivate, and for the combination of all trimming and resampling procedures. This resulted in 7 combinations for each data type.

As CRQA does not require matching datapoints no processing was necessary and %REC, %DET, and %LAM were directly calculated from the recurrence plots (i.e., 3 analyses per data type).

Results of the conventional analyses are reported as correlation coefficients r (continuous data) and V (categorical data). The recurrence parameters are described above.

Additionally, we report a Delta value calculated as the distance of the difference between the average correlation of time series of equal length (s_original with itself, but different of the noise term) compared with the specific trimmed case, divided by the range of the scale of the respective correlation coefficient.

### Study 2: Application of CRQA to the sleep heart health study

#### The Sleep Heart Health Study and Sample construction for the current analysis

To investigate the relevance of USCs recurrence patterns for mortality we used data from the Sleep Heart Health Study (SHHS;^16,17^; *n* = 6697) as downloaded from the National Sleep Research Resource in 2020^27,38^. The SHHS was designed to investigate the impact of sleep disturbances (mainly sleep apnea) on cardiovascular health^16^. Patients with treated sleep apnea were excluded but snorers were purposefully oversampled^16^. Sleep was measured using in-home polysomnography during one baseline measurement night. In sum, the SHHS is one of the rare large-scale datasets including polysomnographic sleep assessment.

From the baseline data collection of the SHHS, which took place between December 1995 and January 1998, we extracted an elderly sample of at least 60 years of age and with at least two USCs per night (which is a “conditio sine qua non” for the calculation of CRQA between USCs). This resulted in a sample of *n* = 2248 individuals (age = 72.1 ± 7.11, male = 1068 (47.5%), further information in Table 3).

#### Sleep assessment

In-home polysomnography comprised EEG (C3/A1 and C4/A2), binocular electrooculography (EOGs), submental electromyography (EMG), monitoring of movements of chest and abdomen (via inductive plethysmography bands), airflow, pulse oximetry, ECG, and body position^17^. PSG measurements were manually scored by trained raters according to Rechtschaffen and Kales criteria^24^. Sleep stages S3 and S4 were summed up to one “slow-wave sleep” (SWS) parameter. There was an “excellent”^39^ intra- and interrater reliability regarding the scoring of sleep stages (kappa statistics > 0.80).

#### Extracting USC from polysomnography data

Conventionally, a USC is defined as ranging from the onset of NREM sleep (either at the beginning of the night or after a REM sleep episode) to the offset of the following REM episode. The specific operationalization, however, differs between studies^11,40–43^. The definitory difference concerns mainly the question whether a minimum duration of being in one sleep stage is required to count as a separate USC (it is for instance a frequent occurrence that REM-sleep is interrupted by individual Non-REM-sleep epochs and clearly not every single interruption can be counted as initializing a new sleep cycle). In the present case we defined two REM episode as belonging to different USC when they were at least 20 min apart.

#### Calculating cross-recurrence patterns of USCs

CRQA patterns of USCs were calculated using the algorithm presented in the present manuscript. As an example, in order to extract the total recurrence of between the first USC and second USC using CRQA, the following parameters were used: embedding dimension = 1; delay = 1; Euclidean norm, a very small radius parameter value close to 0 so that different categories were not accidentally counted as recurrent. Moreover, we used standard settings that are conventionally used for the analysis of nominal sequences and that are not of interest here. Below is the R-code to run the function that accompanies this manuscript:

*asymMdcrqa(USC2*,*USC1*,*emb = 1*,*del = 1*,*norm=”euc”*,*rad = 0.0001*,*dline = 2*,*vline = 2*,*zscore = 0*,

*metric=”euclidean”)*

Note that the R-function presented above contains several parameters. A description of the parameters can be found in Coco et al.^44^. In the present context, we are working with categorical data, where each data point indicates the presence of a specific value in the time series. In this case, the parameters are set to their default values without embedding, because changing these parameters will produces mixtures of categories or arbitrarily group together categories that are actually different. For further explanation of differences between applications to continuous and categorical data, see, for example, Wallot^45^.

CRQA was conducted pairwise for all unequal USCs of one night. From these CRQA calculations the standard CRQA parameters total recurrence, determinism, maximal diagonal length, average diagonal length, and entropy were extracted. The results of the pairwise calculations where then merged into average values across all USCs of one night resulting in one average value for each CRQA parameter. Additionally, the results of the CRQA of USC 1 vs. USC 2 were treated separately. This was done since physiological deep sleep is particularly present in the first USC but to a much lesser degree in the subsequent USC. Disturbed sleep has been shown to lead to a convergence of the sleep stages in USC 1 and 2^12,13^. We therefore assumed that the recurrence patterns between USC 1 and USC 2 might be of special importance.

To reduce the number of CRQA parameters we conducted principal component analysis using the PCA function of the FactoMineR package in R^46^. The first five PCA dimension preserved 94.7% of the initial information. When calculating crude Cox-regressions, PCA dimension #3 was significantly associated with increased mortality (Hazard ratio: 1.09 (95% CI 1.02,1.16) and was used for further analysis.

This dimension was maximal positively correlated with the maximal diagonal length between USC 1&2 (*r* = 0.6, Pearson correlation; Figure S1) and best negatively correlated with mean %determinism (i.e., the percentage of total recurrence presenting in diagonal line structures of at least two adjacent recurrence points; *r*= −0.5; Figure S1). Both, determinism and maximal diagonal length are metrics of recurrent sleep stage trajectories (i.e., diagonal line structures) both can be seen as a measure of stability of sleep trajectories between USCs. Thus, we chose to refer to this PCA dimension as “CRQA-stability parameter”. (Note, other parameters such as total recurrence or entropy, cannot readily be seen as reflecting stability of sleep trajectories since the former does not require the recurrence of line structures (i.e., trajectories) and the latter is determined by the distribution of diagonal lengths. That means that both, the recurrence of predominantly short trajectories (unstable sleep) as well as predominantly long trajectories (stable sleep) could result in the same entropy level)

#### Covariates

We adjusted for polysomnographic sleep parameters, sociodemographic and self-reported health parameters. Polysomnographic covariates were selected based on previously reported associations between polysomnographic sleep parameters and all-cause mortality^28^. Thus, we included, total sleep time, REM sleep, S1 sleep, and apnea-hypopnea-index (AHI). The AHI is the sum of apneas and hypopneas per hour of sleep. In line with guidelines for scoring respiratory events^9^, hypopneas were defined by a > 30% flow reduction and > = 3% oxygen desaturation or—alternatively—leading to a subsequent arousal. For sensitivity analysis regarding relevance of sleep apnea/sleep disordered breathing (SDB), we classified SDB into severity groups in accordance with guidelines^47^ and as previously done for the SHHS^48^, namely: (1) No SDB: AHI < 5/h; (2) Mild SDB: AHI 5 to < 15/h; (3) Moderate SDB: AHI 15 to < 30/h; and (4) Severe SDB: AHI > 30 /h.

Other covariates comprised: gender (male, female) and race [white (1), black (2), other (3)], body mass index (BMI; kg/m^2^), and self- reported health. The latter was assessed by the SF-36^49^. The SF-36 is a widely used instrument that measures eight dimensions of health, i.e., physical functioning, bodily pain, role limitations due to physical health problems, role limitations due to personal or emotional problems, general mental health, social functioning, energy/fatigue, and general health perceptions. Scores for each sub-scale range from 0 to 100. Higher scores represent better health status. The individual scales can be merged into a mental health component and a somatic health component. For the sensitivity analysis regarding the time component in the Cox proportional hazards model, age was used as a covariate if time on study was used as time component in the Cox-regression.

#### Data analysis approach

The aim of the second study was to assess the association of CRQA patterns of USCs with all-cause mortality in an elderly sample. First, we obtained descriptive statistics regarding average number and duration of USCs, the average within subject difference in duration between the longest and shortest USC as well as standard deviations of within subjects USC duration.

The main analysis consisted of adjusted Cox proportional hazards regression analysis^50^. Cox proportional hazards analysis^50^ is widely used in epidemiological research to assess the association between one (or more) predictor variables and the occurrence of a certain event (e.g., death) over time. This type of analysis provides hazard ratios (HR), that is, the ratio of the hazard rates (i.e. the frequency of event occurrence) between two groups (e.g., one exposed to a certain exposure, whereas the other is not).

In the Cox models, the CRQA-stability parameter was used as independent variable, all-cause mortality as dependent variable and age as time component. We adjusted for total sleep time, % of REM sleep, % of S1 sleep, apnea-hypopnea-index (AHI), gender, race, BMI, mental and somatic health. No variable selection technique was employed.

Additionally, we conducted two sensitivity analyses regarding (1) the relevance of sleep disordered breathing/sleep apnea and (2) the relevance of age as time component in the Cox regression model. Regarding the relevance of sleep disordered breathing, we conducted the analysis in a subsample with no sleep disordered breathing (SDB, AHI < 5/h), mild SDB (AHI between 5/h and 15/h), and moderate to severe SDB (AHI > 15/h). As for the second sensitivity analysis, there is a current debate whether age or time on study should be used as time component in Cox regression models in observational studies^25,26^. To exclude the possibility of introducing a relevant bias by choice of time component, we re-calculated our primary analysis with time on study as time component and age as a covariate.

Finally, to check plausibility and generalizability of our results, we run the adjusted Cox regression without the CRQA-stability parameter to see whether previously reported polysomnographic predictors of mortality^28^ would reach significance in our sample, too.

Results of the Cox models are reported as Hazard Ratios [95% CI] and p-values. P-values < 0.05 were considered statistically significant. We did not correct for multiple testing for theoretical and practical reasons: First, the concept of correcting for multiple testing based on testing frequency is debated in the statistical community and has been argued to be logically flawed (e.g., Sjölander & Vansteelandt^51^). Second, as mentioned above, the here presented application of CRQA to study sleep-related mortality should be considered a first-of-a-kind *explorative* analysis. Under this circumstances it seems appropriate to focus on not rejecting possible effects prematurely even if it means to potentially increase the risk of type-I-errors. All analysis were done using R Version 4.2.1.

## Electronic supplementary material

Below is the link to the electronic supplementary material.

Supplementary Material 1

## Data Availability

The sleep recurrence patterns were calculated on the Sleep Heart Health Study –Dataset^16^; 10.25822/ghy8-ks59*).* The dataset was retrieved from the National Sleep Research Resource^27^ where it was first published on October 23, 2013.

## Code availability

The R-Code for implementing the CRQA routine and the simulation of the synthetic data can be found in the online supplementary materials (see online repository for this article: https://osf.io/eygzj/).
