# Supplementary material for: Using cross-recurrence quantification analysis to compute similarity measures for time series of unequal length with applications to sleep stage analysis
Source: Sci Rep. 2024 Oct 4;14:23142. doi: 10.1038/s41598-024-73225-x (PMC11452724; doi:10.1038/s41598-024-73225-x)
Supplement: Supplementary file 1 — Supplementary Material 1 [file 41598_2024_73225_MOESM1_ESM.docx]

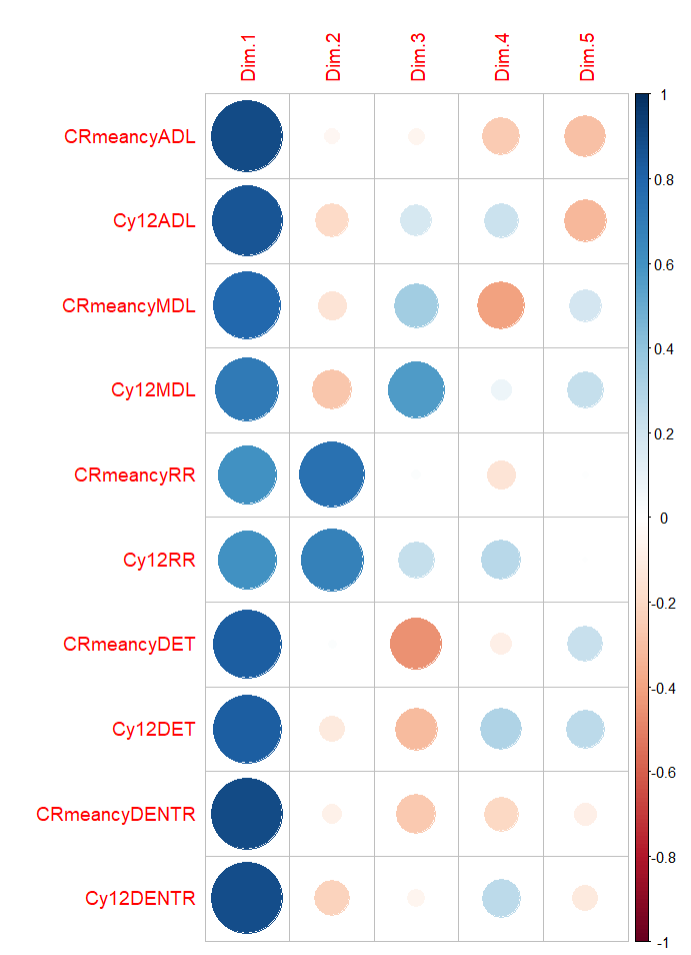


**Figure S1: Correlations between original CRQA parameters and PCA dimensions**

Abbreviations: CRmeancyADL: mean average diagonal length across all pairwise CRQA calculations of one individual. Cy12ADL: average diagonal length between USC 1 and 2. CRmeancyMDL: maximum diagonal length length across all pairwise CRQA calculations of one individual. Cy12MDL: maximum diagonal length between USC 1&2. CRmeancyRR: total recurrence across all pairwise CRQA calculations. Cy12RR: total recurrence between USC 1&2. CRmeancyDET: determinism across all pairwise CRQA calculations. Cy12DET: determinism between USC 1&2. CRmeancyDENTR: entropy across all pairwise CRQA calculations. Cy12DENTR: entropy between USC 1&2. Test: Pearson correlations.
